# Evaluating methodological approaches to assess the severity of infection with SARS-CoV-2 variants: scoping review and applications on Belgian COVID-19 data

**DOI:** 10.1186/s12879-022-07777-6

**Published:** 2022-11-11

**Authors:** Marjan Meurisse, Herman Van Oyen, Koen Blot, Lucy Catteau, Ben Serrien, Sofieke Klamer, Emilie Cauët, Annie Robert, Nina Van Goethem

**Affiliations:** 1 Department of Epidemiology and Public Health, Sciensano, Brussels, Belgium; 2 IREC – EPID, Université Catholique de Louvain, Bruxelles, Belgium; 3 Department of Public Health and Primary Care, Ghent University, Ghent, Belgium

**Keywords:** SARS-CoV-2 variants, Virulence, COVID-19, Surveillance, Belgium, Causal inference, Bias

## Abstract

**Background:**

Differences in the genetic material of severe acute respiratory syndrome coronavirus 2 (SARS-CoV-2) variants may result in altered virulence characteristics. Assessing the disease severity caused by newly emerging variants is essential to estimate their impact on public health. However, causally inferring the intrinsic severity of infection with variants using observational data is a challenging process on which guidance is still limited. We describe potential limitations and biases that researchers are confronted with and evaluate different methodological approaches to study the severity of infection with SARS-CoV-2 variants.

**Methods:**

We reviewed the literature to identify limitations and potential biases in methods used to study the severity of infection with a particular variant. The impact of different methodological choices is illustrated by using real-world data of Belgian hospitalized COVID-19 patients.

**Results:**

We observed different ways of defining coronavirus disease 2019 (COVID-19) disease severity (e.g., admission to the hospital or intensive care unit *versus* the occurrence of severe complications or death) and exposure to a variant (e.g., linkage of the sequencing or genotyping result with the patient data through a unique identifier *versus* categorization of patients based on time periods). Different potential selection biases (e.g., overcontrol bias, endogenous selection bias, sample truncation bias) and factors fluctuating over time (e.g., medical expertise and therapeutic strategies, vaccination coverage and natural immunity, pressure on the healthcare system, affected population groups) according to the successive waves of COVID-19, dominated by different variants, were identified. Using data of Belgian hospitalized COVID-19 patients, we were able to document (i) the robustness of the analyses when using different variant exposure ascertainment methods, (ii) indications of the presence of selection bias and (iii) how important confounding variables are fluctuating over time.

**Conclusions:**

When estimating the unbiased marginal effect of SARS-CoV-2 variants on the severity of infection, different strategies can be used and different assumptions can be made, potentially leading to different conclusions. We propose four best practices to identify and reduce potential bias introduced by the study design, the data analysis approach, and the features of the underlying surveillance strategies and data infrastructure.

**Supplementary Information:**

The online version contains supplementary material available at 10.1186/s12879-022-07777-6.

## Background

Severe acute respiratory syndrome coronavirus 2 (SARS-CoV-2), causing coronavirus disease 2019 (COVID-19) and responsible for a worldwide public health crisis, evolves continuously via genetic changes (e.g., mutations), resulting in an expanding phylogenetic diversity [1]. It has been shown that differences in the genetic material of the virus may result in altered virulence characteristics [2–4]. These genetic changes can be detected with Next Generation Sequencing (NGS) techniques, used to sequence the entire genome (i.e., Whole Genome Sequencing, WGS) or targeted regions of the genome of the virus. Virus variants can be differentiated based on one or more genetic mutations. When a virus variant is already identified, it can also be detected using so-called presumptive genotyping methods, such as S-gene sequencing or PCR screening targeting specific single nucleotide polymorphisms (SNPs), insertions or deletions (i.e., probe detection). Continuous monitoring of circulating variants and assessment of emerging variants is the aim of a genomic surveillance system. The World Health Organization (WHO), in collaboration with the European Centre for Disease Prevention and Control (ECDC) and other partners, defines Variants Of Concern (VOCs) based on (1) the presence of genetic alterations that are expected to alter viral characteristics like transmissibility, immune escape, disease severity or effectiveness of diagnostic or therapeutic tools, and (2) the transmission that influences the prevalence in the population [5, 6]. Since the beginning of the pandemic, different VOCs (Alpha, Beta, Gamma, Delta and Omicron) have been circulating [5, 7]. These VOCs present an increased risk for public health and should be prioritized in public health research. A timely assessment of the disease severity caused by newly emerging variants is essential in order to mitigate the impact on the healthcare system through appropriate public health measures and to provide timely recommendations for policy making and healthcare (e.g., hospital surge capacity preparedness strategies).

Randomization of exposure to a certain SARS-CoV-2 variant, as one would do in a randomized controlled trial (RCT), is not conceivable, making the assessment of severity of infection with variants reliant on observational data. These ‘real-world’ data sources often result from the secondary use of routine care data, such as registries, surveys, medical records, insurance claims data, or government databases. As opposed to data of randomized studies conducted under controlled conditions, observational data are generally full of interactions and biases [8]. However, to identify a causal relationship between SARS-CoV-2 variants (exposure) and COVID-19 disease severity (outcome), we need exposure groups that are similar on both known and unknown factors that may differ between subjects and affect the relationship between the exposure and outcome. This can be referred to as exchangeability of exposure groups (i.e., when the unexposed group is a good approximation for the disease experience of the exposed group had they not been exposed) [9]. When exchangeability of exposure groups cannot be obtained by design, it can still be pursued through statistical methods (e.g. adjustment, matching, inverse probability weighting), thereby mimicking randomization. However, assessing the severity of SARS-CoV-2 variants using observational data in a causal research framework is a challenging endeavor, on which guidance is still limited. Reliability of real-world evidence depends strongly on the quality of the data, assumptions on potential confounding and statistical procedures used.

In this manuscript, we describe potential limitations and biases that researchers can be confronted with when studying the severity of infection with SARS-CoV-2 variants. Further, we evaluate the impact of different methodological choices by analyzing data of Belgian hospitalized COVID-19 patients. Finally, best practices to identify and reduce potential bias introduced by the design of the study and the data analysis approach, or related to the underlying surveillance strategies and data infrastructure, are proposed.

## Methods

A scoping review was conducted to (1) summarize methodological approaches used to study the severity of SARS-CoV-2 variants within different countries using observational data and (2) identify limitations and potential biases resulting from the study design, data analysis approach, underlying surveillance strategies, or data infrastructure. An electronic search was conducted in the PubMed database for the period of the 1st of March 2020 until the 22nd of June 2022, using the following search string: ("SARS-CoV-2 variants" [Supplementary Concept] OR "SARS-CoV-2 variant*" [TIAB] OR "Severe Acute Respiratory Syndrome Coronavirus 2 Variant*" [TIAB] OR "SARS-CoV-2 lineage*" [TIAB] OR "alpha variant"[TIAB] OR "delta variant"[TIAB] OR "beta variant"[TIAB] OR "gamma variant" [TIAB] OR "omicron variant" [TIAB]) AND ("Virulence" [Mesh] OR "virulence" [TIAB] OR "sever*" [TIAB] OR "pathogenic*" [TIAB] OR "death" [TIAB] OR "hospital*" [TIAB] OR "mortality" [TIAB] OR "fatal*" [TIAB] OR "complication*" [TIAB] OR "natural course" [TIAB] OR "Virulence" [Mesh] OR "Severity of Illness Index" [Mesh]) AND ("Observational Study" [Mesh] OR "observational stud*" [TW] OR "Cohort Studies"[Mesh] OR "cohort stud*" [TW] OR "Case–Control Studies" [Mesh] OR "case–control stud*" [TW] OR "Cross-Sectional Studies" [Mesh] OR "cross-sectional stud*" [TW] OR "data-linkage stud*" [TIAB]). We limited the search to English articles focusing on human populations. A first screening phase based on titles and abstracts was conducted [MM], and out-of-topic articles were excluded. A second screening stage based on the full texts was conducted in duplicate by two independent reviewers [MM, NVG]. Articles without a full text available were excluded, in addition to reviews, meta-analyses, non-research evidence (e.g., guidelines, websites, reports, policy documents letters), case reports and preprints. Studies not using observational data for their analyses, only focusing on vaccine effectiveness, not doing statistical inference (only descriptive, no exposure groups), not considering COVID-19 disease severity as an outcome or SARS-CoV-2 variants as a risk factor were also excluded. Discrepancies between the two reviewers were solved by discussion. Additional articles of interest were identified by hand-searching and researching reference lists of selected articles. Information on the following aspects was extracted from included articles in tabular format: (1) first author, (2) year and (3) journal of publication, (4) DOI, (5) country in which the study was conducted, (6) SARS-CoV-2 variants under study (exposure groups), (7) exposure ascertainment (method for variant determination), (8) classification level of viral genomic variation, (9) outcome ascertainment (definition of disease severity), (10) study population, (11) study period, (12) reported potential selection biases following selection of samples for variant determination (13) other potential selection biases reported, (14) confounding factors taken into account, (15) other reported biases or challenges (not related to selection bias), and (16) main conclusion of the study.

The impact of different methodological choices or indications of the presence of biases as identified through the literature search were subsequently illustrated using example data originating from the COVID-19 surveillance in Belgium. A conceptual causal framework to evaluate the effect of SARS-CoV-2 variants on disease severity in a population of hospitalized patients, the corresponding Directed Acyclic Graph (DAG) explicitly stating causal assumptions, and data requirements were described elsewhere [10]. This previous work includes a description of the data infrastructure, allowing individual-level data linkage of selected variables from existing Belgian COVID-19 registries and compliance with the identified data requirements. The linkage of data on hospitalized COVID-19 patients [11], COVID-19 test results (including sequencing information) [12], administered COVID-19 vaccines, and socio-economic indicators was executed through the national registry number.

## Results

We identified 281 articles using the indicated search criteria. 65 articles were selected through the first screening phase (title and abstract screening), 50 articles were finally included based on full text screening, and 6 additional articles were identified through hand-searching and searching of reference lists of included articles (Preferred Reporting Items for Systematic Reviews and Meta-Analyses (PRISMA) flow diagram in Additional file 1: Fig. S1). The completed extraction form describing the characteristics of all included studies is available in Additional file 2.

The results have been categorized into four sections highlighting different aspect of the methodological considerations when studying severity of infection with a SARS-CoV-2 variant. In “Section I: Defining COVID-19 disease severity”, we describe various definitions of COVID-19 disease severity. In “Section II: exposure ascertainment”, we focus on the different SARS-CoV-2 variant exposure ascertainment methods. In “Section III: potential selection biases”, potential selection biases are described. In “Section IV: Factors fluctuating over time needed to be taken into account when studying successive waves of COVID-19 dominated by different variants”, we present a non-exhaustive list of factors fluctuating over time that need to be taken into account when studying successive waves of COVID-19 dominated by different variants.

### Section I: defining COVID-19 disease severity

The severity of a COVID-19 infection can be defined based on several criteria and within different study populations. Within the general population, severity can, among others, be approached as a visit to a general practitioner or the emergency department [13, 14], hospitalization [4, 14–18], intensive care unit (ICU) admission [17–19] or death [18, 20–22]. Other studies may consider the hospitalized population as the population group of interest and may define severity based on the need for ICU admission [2] or the occurrence of death [2, 23], but also based on characteristics of the hospital care, e.g., respiratory or organ support [2, 23]. A distinction can be made between, on the one hand, objective measures of disease severity, such as the monitoring of laboratory biomarkers and “hard” outcomes such as death, and, on the other hand, classifying disease severity based on the patient’s healthcare trajectory, which depends on contextual factors (e.g., clinical interpretations and decisions based on the patient’s condition or pre-existing comorbidities, therapeutic guidelines, or available medical care). The WHO proposed the WHO Clinical progression Scale, ranging from 0 to 10 and based on a patients progress through the healthcare system, as a measure for COVID-19 disease severity that can be used for a broad range of studies and enables comparability between studies [24].

The disease severity outcome definition may determine both the size and direction of the effect measure. Furthermore, risk factors may differ between alternative disease severity outcomes. For example, risk factors for severe complications, such as acute respiratory distress syndrome (ARDS) or death, may be different from those that determine whether a patient is admitted to the ICU. Nursing home residents for instance have characteristics increasing their risk for severe complications (e.g., old age, comorbidities), while clinicians might not be in favor of admitting these patients to the ICU as to prevent disproportionate care [25]. As such, it is important to specify in advance the disease severity outcome(s) of interest and to maintain awareness that conclusions for one outcome might not be transferable to another.

### Section II: exposure ascertainment

Three approaches to define the exposure to a SARS-CoV-2 variant within observational studies were observed in literature: individual-level linkage through a unique identified of (1) WGS or (2) presumptive genotyping results, or (3) categorization of patients based on time periods.

#### Exposure ascertainment based on whole-genome sequencing

The exposure to a variant is ideally confirmed through WGS of the viral isolates obtained from the clinical sample and the subsequent linkage of the obtained sequencing results (e.g. the Pangolin lineage) with the patient data through a unique identifier. However, most countries do not exhaustively perform WGS on all COVID-19 positive samples, but only on a proportion of these samples (see “Section III”), resulting in limited sample sizes when studying severity of infection with a variant. Moreover, a secured data infrastructure needs to be in place to enable the subsequent linkage of the sequencing results with clinical and epidemiological data on the individual patient-level. An example of this study set-up is the observational cohort study in Denmark conducted by Bager et al*.* [26], for which they linked SARS-CoV-2 genomic data with administrative Danish Health Registers in order to estimate the risk of hospital admission in individuals with the Alpha variant compared with those with other SARS-CoV-2 lineages. As such, the study population was restricted to the proportion of cases to which viral genome data could be linked. Given the high WGS capacity in Denmark, this corresponded to 60% of all individuals diagnosed with SARS-CoV-2 during the study period.

#### Exposure ascertainment based on presumptive genotyping methods

A second approach to define exposure to a variant is to use presumptive genotyping methods. For example, Wolter et al*.* [15] assessed the clinical severity of infections with the omicron variant in South-Africa using S gene target failure (SGTF) on the Thermo Fisher Scientific TaqPath COVID-19 PCR test as a proxy. Patients were classified into SGTF or non-SGTF exposure groups based on the identification of the His69_Val70del in the spike protein. However, this can only be accomplished when the TaqPath COVID-19 PCR test was used. Moreover, using SGTF as a proxy for Omicron infections should be limited to well-defined time periods as otherwise other variants also harboring the His69_Val70del, such as Alpha, may be misclassified as Omicron. Also, the Omicron BA.2 sublineage does not contain the His69_Val70del and is therefore not identifiable by SGTF.

#### Exposure ascertainment based on time periods

A third approach to define exposure to a variant is the categorization of patients based on time periods with known variant circulation. This approach does not require the linkage of viral genome sequencing results. Abdullah et al*.* [2] were able to rapidly report the decreased severity of COVID-19 disease in the Omicron-driven fourth wave by comparing the clinical profile of patients admitted at a large hospital in South-Africa. As an advantage, sample sizes are not limited by the linkage to non-exhaustive sequencing or genotyping results and the study population does not suffer from selection bias resulting from a non-random selection of samples (see “Section III”) to determine the viral genomic profile. However, accurately defining the exposure groups highly depends on a representative genomic surveillance system to accurately monitor circulating variants in place and time. This approach may lead to misclassification of variants, especially when the studied time periods are relatively close to each other or in periods with co-circulating variants, possibly diluting the observed effects [13].

#### Illustration of the impact of different exposure ascertainment methods

**Fig. 1:**
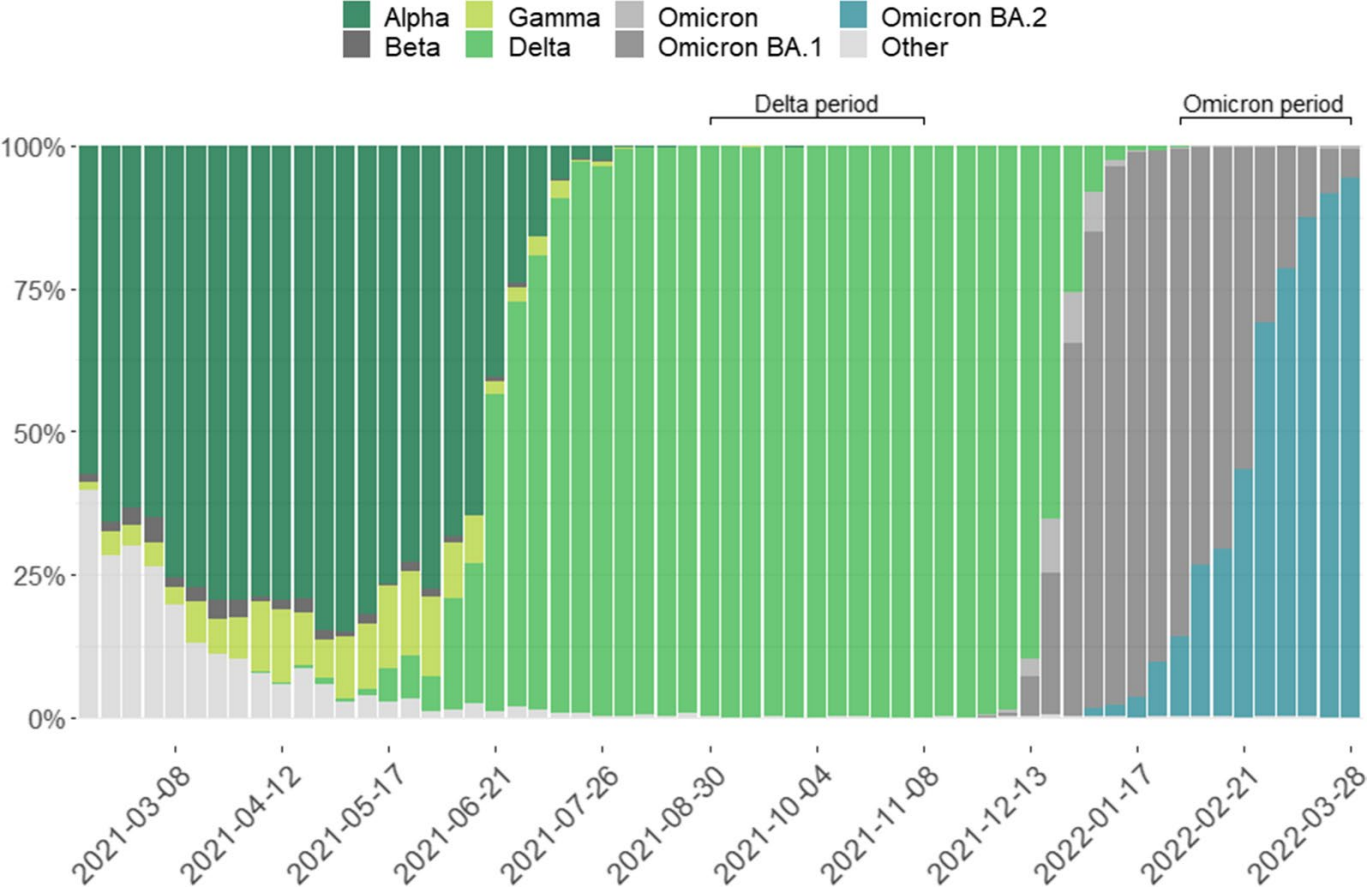
Share of SARS-CoV-2 variants of concern (VOC: Alpha, Beta, Gamma, Delta, Omicron (sub-lineage not specified), Omicron BA.1, Omicron BA.2, and other) per week (x-axis labels indicating the first day of the week) in Belgium as identified though the baseline genomic surveillance. An indication of the exposure groups (Omicron *versus* Delta variant) by time period is given: Delta period 30th of August–14th of November 2021 and Omicron period 31st of January–28th of March 2022

Belgian data of hospitalized COVID-19 patients were used to illustrate the impact of the different exposure ascertainment approaches. The clinical severity of infection with the Omicron variant as compared to the Delta variant has previously been assessed among hospitalized COVID-19 patients in Belgium [27], where severity was defined as being hospitalized for COVID-19 and either experiencing an ARDS event, and/or being admitted to ICU and/or in-hospital mortality. The main analysis was restricted to hospitalized patients registered in the Clinical Hospital Survey (CHS) and with a confirmed (based on WGS) or compatible (based on presumptive genotyping) Omicron or Delta infection as obtained through the linkage with the COVID-19 TestResult database. In addition, a sensitivity analysis was performed, only including patients with a WGS-confirmed Omicron or Delta infection [27]. Here, as an illustration, we apply the same analysis to a study population where the exposure groups were defined based on the date of hospital admission during restricted time periods with an estimated 100% circulation of the Omicron or Delta variant, respectively. These time periods were defined based on the representative baseline genomic surveillance in the Belgian general population [28]. The Delta exposure group was defined as those patients being admitted to the hospital between the 30th of August 2021 and the 14th of November 2021, while the Omicron exposure group was defined as those patients being admitted to the hospital between the 31st of January 2022 and the 28th of March 2022 (see Fig. 1).

**Fig. 2:**
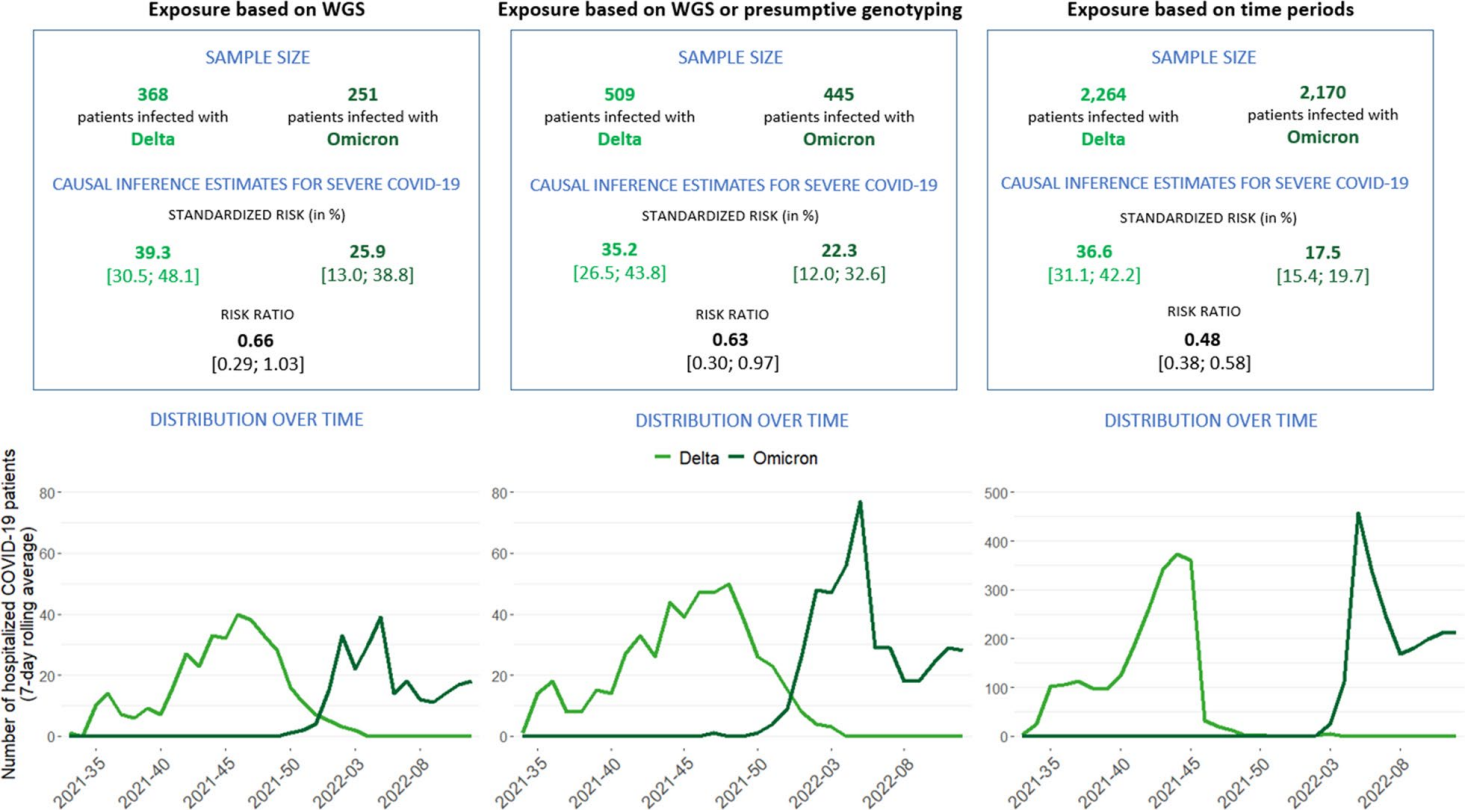
Illustration of different approaches for SARS-CoV-2 variant exposure status ascertainment: (1) exposure defined based on WGS, (2) exposure defined based on WGS or presumptive genotyping and (3) exposure defined based on time periods with known variant circulation. The different approaches are compared in terms of the obtained sample sizes, the inference estimates and their 95% confidence intervals (expressed as standardized risk and risk ratio) for severe COVID-19 (experiencing an acute respiratory distress syndrome (ARDS) event, being admitted to the intensive care unit (ICU), or in-hospital mortality), and the distribution over time of the number of hospitalized COVID-19 patients included in the analysis by week of diagnosis (7-day rolling average). Please note the scale differences in the y-axis in the distribution plots

All data analyses were conducted on the 11th of April 2022, which is almost five months after reporting the first confirmed Omicron case in Belgium. The sample sizes, distribution of cases over time and the obtained causal inference estimates for severe COVID-19 are presented in Fig. 2 for each variant exposure ascertainment method.

The variant of infection was determined by WGS or genotyping for around 12% of the hospitalized COVID-19 patients registered in the CHS. Hence, the size of the study population is substantially reduced when defining exposure to a variant based on the linkage with viral genomic data. The exposure groups as defined based on the time period were compared with the exposure groups as defined based on the variant information obtained through linkage with the WGS or genotyping information from the COVID-19 TestResult database to identify exposure misclassification (see Table 1). For a limited number of patients categorized based on the time period, the obtained variant information through WGS or presumptive genotyping contradicted the exposure group, leading to potential differential exposure misclassification bias. For example, 0.4% of patients in the Delta exposure group as defined based on the time period were in reality infected with an Omicron variant.

**Table 1:** Contrasting, on the one hand, the SARS-CoV-2 variant exposure status as defined based on time periods with known variant circulation with, on the other hand, the SARS-CoV-2 variant exposure status as defined through linkage with Whole Genome Sequencing (WGS) and presumptive genotyping results in the COVID-19 TestResult database

	Delta based on time period^a^	Omicron based on time period^b^
Conf. or comp. Delta^c^	272 (12.0%)	1 (0.0%)
Conf. or comp. Omicron^c^	8 (0.4%)	249 (11.5%)
Other variant	1 (0.0%)	1 (0.0%)
Unknown	1983 (87.6%)	1919 (88.4%)
Total	2264	2170

^a^30th of August–14th of November 2021

^b^31st of January–28th of March 2022

^c^Conf. = confirmed through WGS. Comp. = compatible, based on presumptive genotyping

When looking at the inference estimates as obtained from the analyses executed with the three different approaches for exposure status ascertainment, we observed the same direction of the effect, i.e., the risk for severe COVID-19 (experiencing an ARDS event, or being admitted to the ICU or in-hospital mortality) was found to be lower for hospitalized COVID-19 patients when infected with the Omicron variant, compared to hospitalized COVID-19 patients when infected with the Delta variant (see Fig. 2). However, the effect size obtained in the study population selected based on the time periods (RR = 0.48) differs from the effect sizes obtained within the study population for which the exposure status was defined based on WGS (RR = 0.66) and/or presumptive genotyping (RR = 0.63). The confidence interval (CI) of the effect size obtained in the study population selected based on the time periods (CI = [0.38; 0.58]) is also narrower than those of the effect sizes obtained through the other two approaches (CI = [0.29; 1.03], CI = [0.30; 0.97]), indicating a higher precision of the former estimate. Furthermore, the estimated standardized risk of severe COVID-19 differs between the different approaches (see Fig. 2). It is important to consider the selected study population (depending on the exposure ascertainment method) across which the marginalization was done when interpreting the standardized risk estimates.

### Section III: potential selection biases

Selection bias is any bias resulting from factors associated with the exposure and/or outcome affecting the selection of the study participants [29]. In the current context, selection bias can be introduced when the subset of samples selected for WGS or presumptive genotyping is not representative of the underlying population. To reduce this bias, an appropriate sampling strategy for sequencing or presumptive genotyping (e.g., ensuring representativeness based on the geographical location, patient demographics, and disease severity) is recommended [30, 31]. However, reaching representative sample selection for sequencing or presumptive genotyping is often challenging in current surveillance programs.

#### Selection bias introduced by conditioning on viral load

There exist technical considerations related to the viral RNA abundance in samples when selecting samples for NGS. In many settings, the only samples routinely available for variant identification will be residual diagnostic samples [32]. The viral load changes dynamically over the course of the infection (highest in the first week following disease onset [33, 34], with a more rapid decline among vaccinated individuals [35]) and between different tissues (highest in specimens from the lower respiratory tract [36]). As such, viral load measurements depend on the clinical specimen and on the timing of sampling in the disease trajectory [37, 38]. Sequencing samples with a low viral load (high cycle threshold (Ct) value) is often not recommended due to a rapid drop in the success rate of obtaining a complete or nearly complete viral genome [39], especially when sequencing capacities are limited and cost-efficiency of genomic surveillance has to be taken into account. For example, in the UK, samples are considered positive when minimal two target genes are amplified with a Ct value of less than 37 when using the TaqPath™ COVID-19 RT-PCR, whereas they are selected for WGS when N gene or ORF1ab target Ct value is less than 30 [40]. Likewise, Coolen et al*.* [41] describe a cut-off Ct value of 30 for samples to be sequenced with a high SARS-CoV-2 genome coverage using a reverse complement PCR technique, while generally a RT-PCR test result is considered positive when multiple target genes have a Ct value less than 35–40 [37]. For S-gene targeted Sanger sequencing, similar viral load cut-offs have been reported [42]. It has been demonstrated that the viral load is associated with the infectiousness [38, 43] and severity of the infection [37, 44–48]. Thus, by selecting samples with a sufficiently high viral load for sequencing or PCR probe detection, we might be selecting samples of COVID-19 cases with a more severe SARS-CoV-2 infection, no longer representative of the target population. In addition, viral loads can differ relatively between different SARS-CoV-2 variants [49–53]. Teyssou et al*.* [49] for example observed higher viral loads for the Delta variant compared to the Beta and historical variants.

**Fig. 3:**
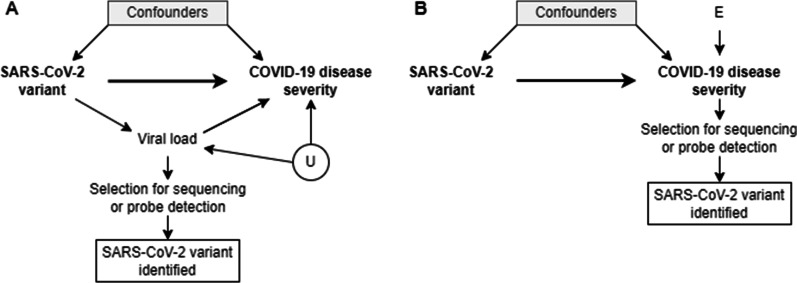
Directed Acyclic Graphs (DAGs) representing the potential selection biases introduced by the selection of COVID-19 cases by the availability of an identified SARS-CoV-2 variant of infection and **A** the selection of samples for variant identification based on the viral load in the sample, or **B** the selection of samples for variant identification based on the disease severity of a patient. Square nodes represent the conditioning on a variable, while circular nodes represent unobserved or unknown variables. U: unmeasured confounders. E: error term. Based on a figure from Van Goethem et al. [10]

The assumptions on the data-generating process and the biological pathways are graphically represented using a DAG in Fig. 3A. Briefly, the causal assumptions encoded in DAGs can identify the variables (minimally sufficient adjustment sets) that we need to control for in order to eliminate confounding, as well as helping to recognize variables that, if controlled for, bias the analysis [54]. Conditioning (also referred to as ‘controlling’ or ‘adjusting’) on a variable can be achieved through either sample restriction, stratification, regression adjustment or matching to examine the association of exposure and outcome within levels of the conditioned variable [55]. More details on DAGs can be found elsewhere [54, 56, 57].

We assume that viral load is a mediator on the causal path between the SARS-CoV-2 variant and COVID-19 disease severity. By selecting COVID-19 cases with an identified SARS-CoV-2 variant of the infection, there is conditioning on a descendant of the viral load (i.e., the mediator), which means there is also conditioning on viral load itself. Conditioning on viral load blocks the indirect path between the exposure and outcome, taking away part of the causal effect of interest. The selection bias which is introduced in this way can be referred to as overcontrol bias [10, 58]. Furthermore, there might be a common unmeasured cause (U) of viral load and COVID-19 disease severity (such as immunosuppression [59, 60]). As viral load is a collider (i.e., a common effect of the treatment—or variable associated with the treatment—and the outcome—or variable associated with the outcome) on the path ‘SARS-CoV-2 variant → Viral load ← U → COVID-19 disease severity’, conditioning on viral load would open a backdoor path and introduce a spurious association between SARS-CoV-2 variant and COVID-19 disease severity. This is a form of endogenous selection bias, introduced by conditioning on an intermediate collider [58].

In order to limit this potential selection bias, in Denmark they aim to sequence the viral genome of all positive samples, with no limitation on the Ct value [61]. However, the chance of successfully obtaining a viral genome remains smaller for samples with a higher Ct value, and as such completely eliminating a selection of samples based on Ct values is not feasible with the current technical limitations. Furthermore, in periods with increased numbers of cases, Denmark’s ambition has been shown not to be reachable and some restriction of samples based on the Ct value were still required [4, 62, 63].

Given the complex relationships that exist between the viral load, SARS-CoV-2 variants and severity of illness (e.g., a modifying effect by age [64]), it is difficult to assess the implications and consequences of selecting samples for sequencing based on the viral load for the subsequent epidemiological analyses.

#### Selection bias introduced by conditioning on COVID-19 disease severity

In most countries, exhaustive sequencing of eligible positive diagnostic samples remains challenging. In Europe, the percentage of sequences generated and shared of reported COVID-19 cases is 2.7% since the 10th of January 2020 [65] (as accessed on the 7th of April 2022), however large differences in sequence coverage are observed between countries [65, 66]. When the sequencing of eligible samples is not exhaustive, the selection might be biased towards those samples of COVID-19 cases with certain characteristics. For example, in a hospitalized cohort, samples of patients with a severe disease outcome might be preferentially selected for variant identification. When assuming that there are some factors (which can be taken together in an error term E) affecting COVID-19 disease severity (see Fig. 3B), the outcome (COVID-19 disease severity) is a collider on the path between the exposure (SARS-CoV-2 variant) and the error term of the outcome (E). As such, selecting samples of COVID-19 patients with a severe disease outcome (i.e., selection on the outcome), opens a non-causal path ‘SARS-CoV-2 variant → COVID-19 disease severity ← E’. As a result, the association between COVID-19 disease severity and SARS-CoV-2 variant does not represent the causal effect of interest, and the internal validity of the study is affected. The bias introduced in this way is referred to as sample truncation bias, a form of endogenous selection bias introduced by conditioning on the outcome (or a descendent of the outcome). [58]. Furthermore, selecting patients on certain patient characteristics (e.g., based on travel history or vaccination status) hampers the external validity or representativeness of the study, by inducing differences between the study population and target population [67].

#### Illustration of study population selection and assessment of potential selection bias

**Fig. 4:**
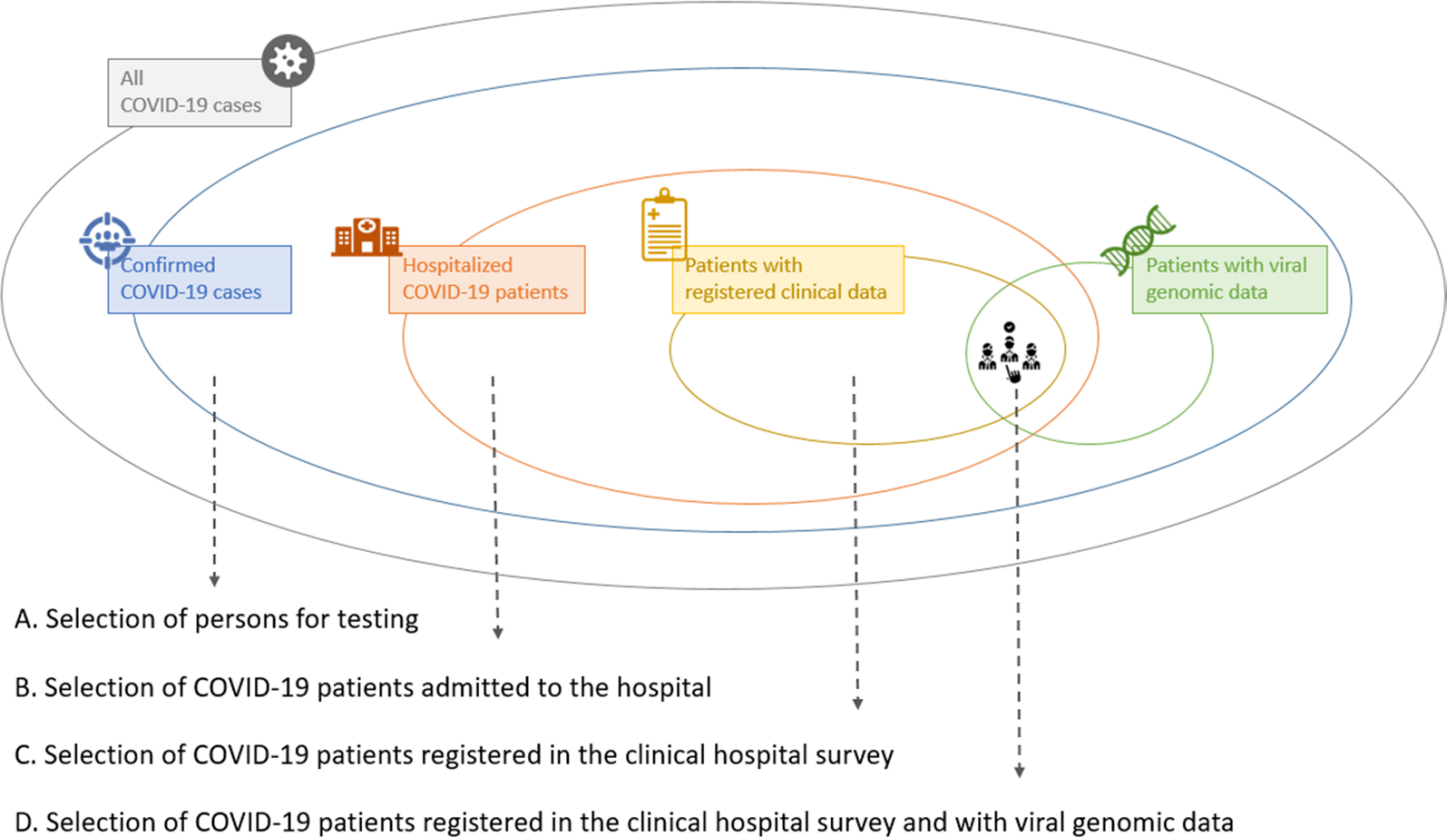
Overview of the different selection steps to obtain the study population of Belgian hospitalized COVID-19 patients registered in the Clinical Hospital Surveillance (CHS). This study population has been used to study the severity of SARS-CoV-2 variants. Selecting study participants can introduce selection bias

The data of Belgian hospitalized COVID-19 patients with variant information results from different selection steps, as visualized in Fig. 4. Firstly, diagnostic COVID-19 testing is based on national testing strategies, designed to set rules for prioritization of testing, and thus not at random (Fig. 4A). The testing strategy in Belgium has changed over time. For instance, testing of asymptomatic high-risk contacts was put on hold at the end of 2021, to decrease the pressure on laboratories [68]. This results in differential selection of patients based on their disease severity, which might change over time. Secondly, the study population is restricted to COVID-19 patients admitted to the hospital (Fig. 4B) and registered in the CHS (Fig. 4C). Again, a selection on the severity of disease in patients is performed, potentially resulting in collider bias and hampering both the internal and external validity of the study [69]. However, the advantage of working within this sub-cohort of hospitalized patients is the availability of detailed clinical information, allowing to adjust for important confounders in the study. Thirdly, for 9.8% of the Belgian hospitalized COVID-19 patients admitted after the 1st of March 2021 and registered in the CHS, the variant of infection was confirmed through WGS (Fig. 4D). However this percentage fluctuates with time. For example, during the third and fourth epidemiological wave (15th of February–27th of June 2021 and 4th of October–26th of December retrospectively) the absolute number of hospitalized patients with variant information confirmed through WGS increases and the percentage coverage decreases, while during the interwave period in between the absolute number decreases and the percentage coverage increases (see Additional file 3: Fig. S2). The Belgian genomic surveillance consists of (1) a baseline surveillance, in which 5–10% of positive COVID-19 samples from sentinel laboratories are requested to be selected at random for WGS and (2) an active surveillance, where samples are requested to be selected for the detection of variants for patients with specific characteristics (i.e., patients with a travel history, re-infection, chronic infection or vaccination breakthrough infection) [70]. A random selection of samples to be sequenced, as in the baseline surveillance, limits potential selection biases. Hence, in this setting it is recommended to select patients with variant information available through baseline surveillance for the study population to assess severity of SARS-CoV-2 variants, which requires a correct reporting of the indication for selection for sequencing.

There are multiple options to assess selection bias when studying the severity of SARS-CoV-2 variants. First, selection of study participants only introduces bias when associated with exposure and/or outcome. Bager et al*.* [4] studied the risk of hospitalization associated with infection with the SARS-CoV-2 Alpha variant compared to other co-circulating variants in Denmark, and they assessed the presence of selection bias by looking at, on the one hand, the relative risk of hospitalization among individuals without variant information relative to a reference group of non-Alpha strains (adjusted RR = 0.96 [0.90; 1.04]) and, on the other hand, the relative risk of hospitalization of individuals with variant info for the Alpha strain (adjusted RR = 1.23 [1.10; 1.38]) relative to the same reference group. They did not observe a strong association between the availability of variant information and hospitalization. Second, selection bias can also be identified by comparing the baseline characteristics of COVID-19 cases with variant info to those without, assessing whether patients selected into the study are representative for the target population (i.e., checking the external validity). This was illustrated using the data of Belgian hospitalized COVID-19 patients, by comparing baseline characteristics between the patients admitted between 1st of March 2021–28th of March 2022 with confirmed (by WGS) variant information available through baseline surveillance and without any variant information available (neither confirmed by WGS, nor compatible, based on presumptive genotyping) (Table 2). Patients with confirmed variant information available through baseline surveillance are slightly older, more frequently have a non-European ethnicity and certain comorbidities compared to patients without variant information available. Furthermore, they were more frequently fully vaccinated with a primary vaccination schedule, admitted to a university hospital and had a severe COVID-19 infection. As such, the study population, selected based on the availability of confirmed variant info through baseline surveillance, differs from and cannot be considered truly representative for the general hospitalized population (i.e., the population of interest). The higher frequency of patients with a severe disease outcome in the study population indicates selection on the outcome variable, and as such the presence of sample truncation bias. Furthermore, selection of samples of COVID-19 patients based on certain patient characteristics (e.g., age, vaccination status, comorbidities) might hamper the generalizability of results.

**Table 2:** Baseline characteristics of Belgian hospitalized COVID-19 patients registered in the Clinical Hospital Surveillance (CHS)

	Hospitalized patients (admitted between 1/3/2021–28/3/2022) with available variant information (confirmed by WGS^a^) from baseline surveillance (n = 1767)			Hospitalized patients (admitted between 1/3/2021–28/3/2022) without any available variant information (n = 28,728)		
		%	n		%	n
*Demographics*						
Age (years), median (IQR)	69 (50–87)		1767	66 (45–80)		28,728
Male gender, n (%)	945	53.5	1765	14,776	51.5	28,714
Nursing home resident, n (%)	109	6.2	1754	1698	6.0	28,347
Ethnicity, n (%)						
European	559	78.2	715	10,631	84.8	12,535
North-African	86	12.0	715	1173	9.4	12,535
Sub-Saharan African	35	4.9	715	322	2.6	12,535
Asian	29	4.1	715	301	2.4	12,535
Hispanic	6	0.8	715	108	0.9	12,535
*Comorbidities*						
Cardiovascular disease, n (%)	622	35.3	1763	8208	28.6	28,663
History of arterial hypertension, n (%)	597	33.9	1763	9529	33.2	28,663
Diabetes mellitus, n (%)	394	22.3	1763	5530	19.3	28,663
Obesity, n (%)	211	12.0	1763	3478	12.1	28,663
Chronic pulmonary disease, n (%)	310	17.6	1763	3916	13.7	28,663
Chronic neurological disease, n (%)	207	11.7	1763	2349	8.2	28,663
Chronic cognitive deficit, n (%)	175	9.9	1763	2400	8.4	28,663
Chronic renal disease, n (%)	332	18.8	1763	3363	13.4	28,663
Chronic liver disease, n (%)	54	3.1	1763	774	2.7	28,663
Solid cancer, n (%)	269	15.3	1763	2884	10.1	28,663
Haematological cancer, n (%)	77	4.4	1763	529	2.1	28,663
Chronic Immunosuppression, n (%)	147	8.3	1763	622	2.2	28,663
*Socio-economic status*						
Education level, n (%)						
Lower	249	25.8	965	3904	25.5	15,299
Lower secondary	290	30.1	965	4644	30.4	15,299
Higher secondary	270	28.0	965	4046	26.4	15,299
Post-secondary higher education	156	16.2	965	2705	17.7	15,299
Population density^b^, median (IQR)	1105 (419–2590)		1738	740 (332–1778)		25,916
Median taxable income per capita^c^, median (IQR)	26,733 (23,807–28,756)		1738	26,787 (23,807–28,352)		25,916
*Exposure*						
Place of infection, n (%)						
Community-acquired	1430	81.6	1752	24,491	86.5	28,302
Hospital-acquired^d^	231	13.2	1752	2302	8.1	28,302
Nursing home-acquired	91	5.2	1752	1509	5.3	28,302
*Vaccination status*						
Vaccination category, n (%)						
Not vaccinated	747	42.3	1767	15,010	52.2	28,728
Partially vaccinated	62	3.5	1767	1080	3.8	28,728
Fully vaccinated	497	28.1	1767	6779	23.6	28,728
Fully vaccinated + booster	461	26.1	1767	5859	20.4	28,728
*Disease characteristics*						
Fever at admission, n (%)	376	50.9	738	6273	48.3	12,983
Viral syndrome at admission, n (%)	322	43.3	744	5727	44.8	12,789
Lower respiratory symptoms at admission, n (%)	520	68.6	758	8908	67.7	13,162
Upper respiratory symptoms at admission, n (%)	69	9.6	716	1449	11.6	12,528
Anosmia at admission, n (%)	56	8.0	703	1040	8.4	12,341
CRP (mg/l) on admission, median (IQR)	95 (41–186)		136	102 (47–168		1387
*Hospital characteristics*						
Type of hospital, n (%)						
General hospital	584	33.1	1767	21,417	74.6	28,728
General hospital with university character	700	39.6	1767	4982	17.3	28,728
University hospital	483	27.3	1767	2329	8.1	28,728
Mean ICU occupancy during hospital stay, median (IQR)	19 (12–32)		1767	27 (15–40)		28,728
*Outcomes*						
Severe COVID-19^e^, n (%)	440	25.2	1748	5840	20.7	28,147
ICU admission, n (%)	298	16.9	1766	3325	11.6	28,722
ICU transfer, n (%)						
General hospital	81	27.2	298	2228	67.0	3325
General hospital with university character	126	42.3	298	677	20.4	3325
University hospital	91	30.5	298	420	12.6	3325
In-hospital mortality, n (%)	230	13.2	1747	3309	11.8	28,131
Invasive ventilation, n (%)	89	34.0	262	1089	42.5	2564
ECLS, n (%)	7	2.7	262	85	3.4	2535
Hospital length of stay (days), median (IQR)	9 (4–21)		1767	7 (3–15)		28,727

^a^Whole genome sequencing

^b^Population density at the place of residence of the patient (postal code level)

^c^Median net taxable income per capita at the place of residence of the patient (postal code level)

^d^Diagnosis or onset of symptoms more than 8 days after hospital admission of the patient

^e^Experiencing an acute respiratory distress syndrome (ARDS) event, being admitted to the intensive care unit (ICU), or in-hospital mortality

### Section IV: factors fluctuating over time needed to be taken into account when studying successive waves of COVID-19 dominated by different variants

Different factors affecting the relationship between SARS-CoV-2 variants and disease severity can fluctuate over time, which is important to consider when studying variants not co-circulating in time. We discuss in more detail a non-exhaustive list of such factors fluctuating over time: (i) medical expertise, therapeutic strategies and guidelines, (ii) vaccination coverage and natural immunity, (iii) pressure on the healthcare system and (iv) affected population groups.

#### Level of medical expertise, therapeutic strategies and guidelines

First, the level of medical expertise and availability of clinical tools has increased throughout the epidemic. As an illustration, COVID-19 mortality scores developed at the beginning of the pandemic are no longer accurate and need to be evaluated regularly according to the changing clinical context [71]. With the emergence of the first VOCs (Beta and Alpha) in the autumn of 2020 [6], clinical knowledge had already increased compared to the first months of the epidemic, with wild-type and other SARS-CoV-2 strains circulating [72–77]. Experience, practical guidelines and recommendations (e.g., for certain therapies) were quickly shared [77]. In the beginning of the epidemic therapies existed mainly of repurposed therapeutics, but currently novel therapeutic options have been developed, among which antiviral medication (e.g., remdesivir, molnupiravir, paxlovid), anti-inflammatory drugs (e.g., dexamethasone) and monoclonal antibodies [78]. However, effectivity of therapeutics and recommended treatments might be altered with newly emerging variants [79, 80]. Hence, when studying the severity of variants separated in time, taking into account evolving medical expertise, guidelines for treatment and availability of clinical therapies can be important to eliminate confounding bias. Data to adjust for this confounding bias are often lacking. Restricting the analysis to a limited time period can increase the homogeneity in medical expertise and available therapeutics between the exposed and unexposed population.

#### Vaccination coverage and natural immunity

An unprecedented vaccine response was seen during the course of the epidemic. Large scale vaccination campaigns have been undertaken across Europe, gradually increasing the vaccination coverage with a primary course to 72.6% [81] (as accessed on the 11th of April 2022) in the total population of reporting countries within the EU and European Economic Area. Vaccination has been shown to be effective in preventing, to various degrees, a symptomatic infection [82–84], hospitalization [82, 85–91], ICU admission [87] and death [82, 89–91]. However, effectiveness of vaccines can change when new variants emerge [92–94]. When comparing the severity of variants circulating in time periods with a different level of vaccine-induced immunity in the population, adjusting for the vaccination status of persons in the study population reduces confounding bias. In addition, different vaccine types, brands and regimens, introduced at different time points, may have an impact on vaccine effectiveness [95, 96].

Next to vaccination, immunity can also be induced by a previous infection with a SARS-CoV-2 virus, referred to as natural immunity [97]. Similarly, the effectiveness of a previous infection to reduce the risk of a reinfection and severe disease outcomes can also differ between variants of reinfection [98, 99]. Altarawneh et al*.* [98] observed that natural immunity was less effective in preventing a reinfection with the Omicron variant, characterized by specific mutations that might enable the virus to evade the immune system of an individual, compared to a reinfection with the Alpha, Beta and Delta variant. To remove confounding bias introduced by variable natural immunity, adjustment for the previous infection of an individual can additionally be done. It is however only possible to adjust for documented previous infections, and depending on the testing strategy, which changes over time, only a fraction of the SARS-CoV-2 infections will truly be captured by routine surveillance (mainly symptomatic infections will be captured).

Due to limited vaccination coverage and natural immunity in the general population during the first year of the epidemic, confounding bias due to these factors might be limited when comparing variants circulating in this time period. However, with large-scale vaccination programs and increased virus circulation due to relaxation of COVID-19 measures after the first year of the epidemic, assessing severity of variants circulating during a later time period requires adjustment for vaccination status and previous infection of individuals to obtain unbiased estimates. It is additionally important to take into account the time since the last dose of vaccination and for reinfection, as vaccine effectiveness and natural immunity wane [99–102].

#### Pressure on the healthcare system

Factors affecting the pressure on the healthcare system, e.g., stocks of medical equipment, surge of cases, hospital load and availability of staff, vary over time. Different epidemiological waves, during which pressure on the healthcare system is high, follow each other and are separated by interwave periods with reduced pressure on the healthcare system. In periods of high pressure on the healthcare system, the delay between the onset of symptoms and diagnosis or hospital admission generally increases [103]. This delay has been shown to be positively associated with the severity of COVID-19 [104–106]. Additionally, hospital and ICU loads increase in epidemiological wave periods, with increased loads associated with increased disease severity and higher in-hospital mortality [107–110] of COVID-19 patients. Ideally, analyses should be adjusted for hospital load [111], ICU load [10, 112] and COVID-19 incidence [111, 113] to eliminate spurious association through these factors.

#### Affected population groups

Patient characteristics of the affected population, like age, gender, ethnicity, socio-economic status and nursing home residence, might also fluctuate over time depending on the outbreak dynamics. Within the hospital setting, as wards or ICUs overcrowd during epidemic waves, a clinical triage and admission of more severely ill patients also occurs, which can lead to a hospitalized patient population with different demographics and clinical outcomes depending on the epidemiological curve.

#### Illustration of the fluctuations of important risk factors over time

The fluctuation over time of important risk factors for severe COVID-19 was illustrated using the data of the Belgian hospitalized COVID-19 patients. By plotting the vaccination status and the presence of a documented previous infection of these patients over time (see Additional file 4: Fig. S3, Additional file 5: Fig. S4), we could observe that the immunity of patients (vaccine-induced and natural) has increased over the course of the epidemic. However, the acquired immunity can wane with time or might be more efficiently evaded by certain variants. Furthermore, the percentage of nursing home residents and median age of hospitalized COVID-19 patients changes over time (see Additional file 6: Fig. S5, Additional file 7: Fig. S6), with in certain periods more vulnerable individuals (nursing home residents, individuals with a higher age) entering the population of hospitalized COVID-19 patients. These individuals are more likely to develop severe outcomes. Lastly, we can see that the ICU occupancy goes up and down with the different epidemiological waves (see Additional file 7: Fig. S6), with the height of the peaks also changing with time. When assessing severity of variants in the population of Belgian hospitalized COVID-19 patients, we took these different factors into account for the estimation of a causal effect in “Section II” (Fig. 2).

## Discussion

Studying the severity of newly emerging SARS-CoV-2 variants is important to assess the potential impact of these variants on patients’ health, medical practice and the healthcare system, and to guide public health response. In this manuscript, we reviewed different methodological choices made and biases accounted for or reported in literature studying the causal effect of SARS-CoV-2 variants on COVID-19 disease severity using observational data, which in turn can lead to different conclusions. We described different COVID-19 disease severity definitions, e.g., admission to the hospital or intensive care unit *versus* the occurrence of severe complications or death. Different ways to define exposure to a SARS-CoV-2 variant were also described, e.g., linkage of the sequencing or genotyping result with patient data through a unique identifier *versus* categorization of patients based on time periods. In a number of the included articles a combination of exposure ascertainment approaches was adopted. One included article reported an additional exposure ascertainment approach, namely, Fillâtre et al. [114] classified high-risk contacts of a COVID-19 case based on the identified variant of infection of this case. Furthermore, we presented potential selection biases (e.g., overcontrol bias, endogenous selection bias, sample truncation bias). Selection based on factors associated with the exposure and/or outcome might result in biased relative effect sizes when guidelines and procedures for sequencing change over time. Non-random selection of study participants also affects the external validity of a study, irrespective of a difference in selection procedures in the compared exposure groups. Lastly, a non-exhaustive list of time-dependent factors that might be present when studying the severity of variants was given (e.g., medical expertise and therapeutic strategies, vaccination coverage and natural immunity, pressure on the healthcare system per wave, affected population groups).

The scoping literature review shows the diversity of methodological decisions made, confounding biases taken into account, and selection biases present and reported. Based on the results of this literature review and experiences from the Belgian COVID-19 surveillance data, we present recommendations in the form of four best practices for making methodological decisions and limiting potential bias.

### Four best practices when studying disease severity of SARS-COV-2 variants

1. Different choices in COVID-19 disease outcome measure might change the size and direction of the causal relationship. It is recommended to define the outcome under study to fit the public health impact that is targeted. For example, when assessing the impact of a new variant on the ICU load in hospitals, admission to ICU can be recommended as disease outcome. While, when assessing the impact of a new variant on the primary care system, visits to a general practitioner might present the most suitable disease outcome. The size and direction of the effect of a variant on a particular COVID-19 disease outcome, as well as the considered confounding factors, may not be transferrable between different outcome measures.
2. The decision on an approach to define exposure to a SARS-CoV-2 variant should be made on a case-by-case basis depending on the context and setting. However, a representative baseline genomic surveillance, independent of any health outcome, is essential to make this decision and is strongly recommended to facilitate research on this topic.
  a When comparing co-circulating SARS-CoV-2 variants, defining exposure to a SARS-CoV-2 variant based on linkage with variant information is recommended, to prevent potential misclassification bias. When limiting the study population to patients with available variant information, a representative sample selection for WGS (or alternatively S-gene sequencing, or PCR probe screening) is necessary in order to limit selection bias.
  b When comparing variants separated in time and with a close to 100% circulation (as obtained from representative genomic surveillance), and when the coverage of COVID-19 patients with available variant information is limited, defining exposure to a SARS-CoV-2 variant based on time of diagnosis is recommended. In this way, larger sample sizes can be obtained and external validity of the study findings can be increased. However, it is important to be aware of the potential misclassification bias. Furthermore, when comparing the severity of variants circulating in strictly separated time periods, it is important to take into account time-dependent factors in the analyses, such as the evolution of immunity status in the population.
  c The choice between (a) and (b) affects the timing of the analysis. Using linkage with variant information allows for a quick analysis based on the first patients when a new variant emerges and may be important to predict upcoming loads on the healthcare system. When the time between emergence of a variant and 100% circulation takes several weeks, an analysis may come too late to predict upcoming load on the healthcare system. The speed with which the analysis must be done should be balanced against sample size requirements and confounding/selection biases that can be controlled for.
3. A representative sampling of the study population (e.g., based on the geographical location, patient demographics, and disease severity) is essential for the external validity of the study and to allow inference of the effects for the target population. When representative sampling is not feasible, it is important to be aware of the potential presence of selection bias and to not make inferences for target populations for which the study population is not representative. DAGs, graphically displaying the knowledge and assumptions about the causal relationship between exposure and outcome, provide a useful tool to identify potential selection biases.
4. When comparing the severity of variants circulating in different time periods, different important time-dependent risk factors need to be considered as potential confounders. It is recommended to determine, based on expert and continuously updating knowledge, which time-dependent factors might be different between exposure groups, and therefore might distort the association of interest.

## Supplementary Information

**Additional file 1: Figure S1.** Preferred Reporting Items for Systematic Reviews and Meta-Analyses (PRISMA) flow diagram, presenting the selection process of articles included in the scoping literature review, conducted to summarize methodological approaches to study the severity of SARS-CoV-2 variants using observational data and identify limitations and potential biases resulting from the study design, data analysis approach, underlying surveillance strategies, or data infrastructure.

**Additional file 2.** Data extraction form.

**Additional file 3: Figure S2.** The number of Belgian hospitalized COVID-19 patients registered in the Clinical Hospital Surveillance (CHS) and admitted between the 1st of March 2021 and the 28th of March 2022 by the availability of SARS-CoV-2 variant information (variant information confirmed by Whole Genome Sequencing (WGS) available *versus* no confirmed variant information available) (left y-axis) and the coverage of Belgian hospitalized COVID-19 patients with confirmed variant information, i.e. percentage of patients with available confirmed (by WGS) viral information, among the of Belgian hospitalized COVID-19 patients admitted between the 1st of March 2021 and the 28th of March 2022 (right y-axis).

**Additional file 4: Figure S3.** Vaccination status of Belgian hospitalized COVID-19 patients registered in the Clinical Hospital Survey (CHS). (Left) Number of Belgian hospitalized COVID-19 patients with a certain vaccination status over time, 7-day rolling average. (Right) Percentage of Belgian hospitalized COVID-19 patients with a certain vaccination status over time, 7-day rolling average. Periods of dominance of SARS-CoV-2 variants (more than 50% presence in baseline surveillance) are indicated as areas on the plot. Not vaccinated = no dose of a vaccine. Partially vaccinated = one dose of the BNT162b2, mRNA-1273 or NVX-CoV2373 vaccine. Fully vaccinated = one dose of the Ad26.COV2.S vaccine, two doses of the BNT162b2, mRNA-1273 or NVX-CoV2373 vaccine, or a mixture of two doses of the latter three vaccines (= primary vaccination schedule). Fully vaccinated + booster = primary vaccination schedule plus an additional dose of the BNT162b2 or mRNA-1273 vaccine.

**Additional file 5: Figure S4.** Documented previous SARS-CoV-2 infections of Belgian hospitalized COVID-19 patients registered in the Clinical Hospital Survey (CHS). (Left) Number of Belgian hospitalized COVID-19 patients with and without a documented previous infections, 7-day rolling average. (Right) Percentage of Belgian hospitalized COVID-19 patients with and without a documented previous infections, 7-day rolling average. Periods of dominance of SARS-CoV-2 variants (more than 50% presence in baseline surveillance) are indicated as areas on the plot.

**Additional file 6: Figure S5.** Nursing home residents within the population of Belgian hospitalized COVID-19 patients registered in the Clinical Hospital Survey (CHS). (Left) Number of Belgian hospitalized COVID-19 patients that are nursing home residents, 7-day rolling average. (Right) Percentage of Belgian hospitalized COVID-19 patients that are nursing home residents, 7-day rolling average. Periods of dominance of SARS-CoV-2 variants (more than 50% presence in baseline surveillance) are indicated as areas on the plot.

**Additional file 7: Figure S6.** (Left) Median age of Belgian hospitalized COVID-19 patients registered in the Clinical Hospital Survey (CHS), 7-day rolling average. (Right) Median of the mean ICU occupancy of Belgian hospitalized COVID-19 patients registered in the CHS, 7-day rolling average. Periods of dominance of SARS-CoV-2 variants (more than 50% presence in baseline surveillance) are indicated as areas on the plot.

## Data Availability

Individual-level data of the Belgian hospitalized COVID-19 patients registered in the Clinical Hospital Surveillance cannot be shared as open data, as they are too detailed to preserve patients’ privacy. Data of the different registries within the LINK-VACC project (Clinical Hospital Survey, COVID-19 TestResult Database, StatBel, and Vaccinnet +) are stored in a secured environment provided by healthdata.be. Linking the individual-level data within this secured environment was possible with to the use of a pseudonymized national reference number. Access to this secured healthdata.be environment was granted ad nominatum to scientists involved in Sciensano’s surveillance activities. The selected data can be requested by external investigators by filling in the data request form (https://epistat.wiv-isp.be/datarequest). When of sensitive nature, the provision of the requested data has to be approved by the Belgian Information Security Committee Social Security & Health, and has to be outlined in a data transfer agreement (DTA) with the data owner (Sciensano).

## Abbreviations

SARS-CoV-2: Severe acute respiratory syndrome coronavirus 2
COVID-19: Coronavirus disease 2019
NGS: Next generation sequencing
WGS: Whole genome sequencing
SNP: Single nucleotide polymorphism
WHO: World Health Organization
ECDC: European Centre for Disease Prevention and Control
VOC: Variant of concern
RCT: Randomized controlled trial
PRISMA: Preferred Reporting Items for Systematic Reviews and Meta-Analyses
ICU: Intensive care unit
ARDS: Acute respiratory distress syndrome
CHS: Clinical Hospital Survey
CI: Confidence interval
Ct: Cycle threshold
DAG: Directed acyclic graph
