# Supplementary material for: Evaluating methodological approaches to assess the severity of infection with SARS-CoV-2 variants: scoping review and applications on Belgian COVID-19 data
Source: BMC Infect Dis. 2022 Nov 11;22:839. doi: 10.1186/s12879-022-07777-6 (PMC9651100; doi:10.1186/s12879-022-07777-6)
Supplement: Supplementary file 1 — Additional file 1: Figure S1. Preferred Reporting Items for Systematic Reviews and Meta-Analyses (PRISMA) flow diagram, presenting the selection process of articles included in the scoping literature review, conducted to summarize methodological approaches to study the severity of SARS-CoV-2 variants using observational data and identify limitations and potential biases resulting from the study design, data analysis approach, underlying surveillance strategies, or data infrastructure. [file 12879_2022_7777_MOESM1_ESM.docx]

#### Additional File 1: PRISMA flow diagram


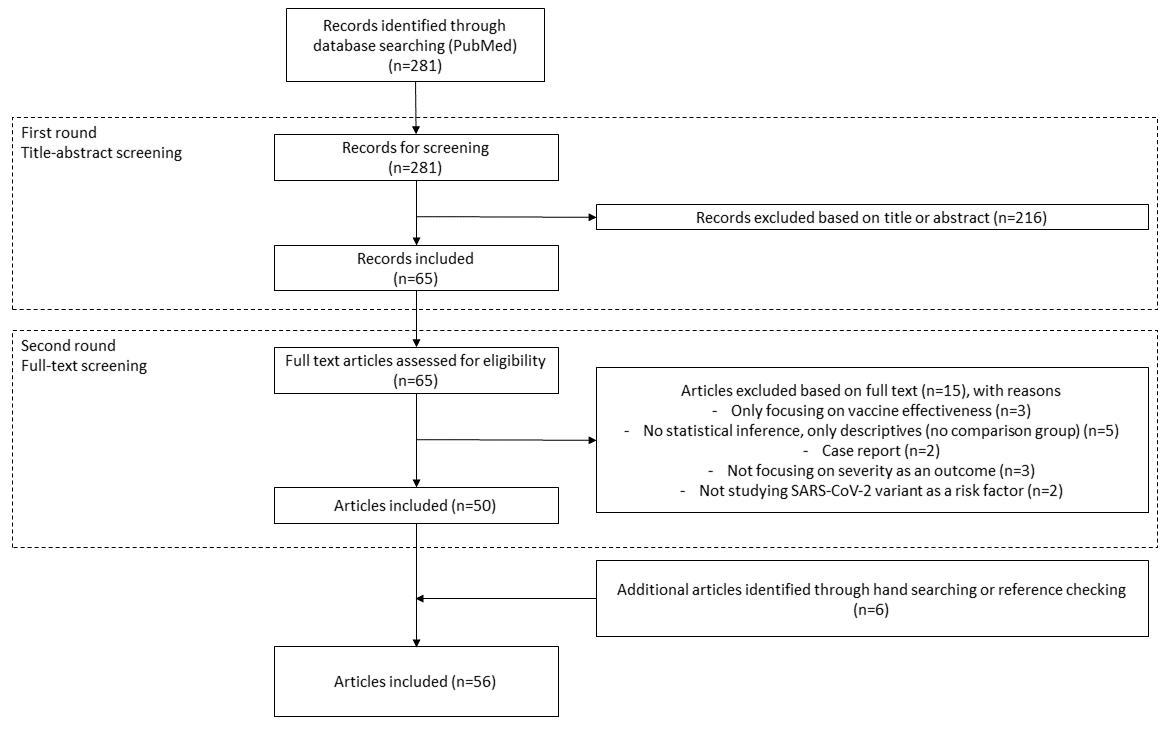


*Figure S1. Preferred Reporting Items for Systematic Reviews and Meta-Analyses (PRISMA) flow diagram, presenting the selection process of articles included in the scoping literature review, conducted to summarize methodological approaches to study the severity of SARS-CoV-2 variants using observational data and identify limitations and potential biases resulting from the study design, data analysis approach, underlying surveillance strategies, or data infrastructure.*
