# Supplementary material for: Evaluating methodological approaches to assess the severity of infection with SARS-CoV-2 variants: scoping review and applications on Belgian COVID-19 data
Source: BMC Infect Dis. 2022 Nov 11;22:839. doi: 10.1186/s12879-022-07777-6 (PMC9651100; doi:10.1186/s12879-022-07777-6)
Supplement: Supplementary file 3 — Additional file 3: Figure S2. The number of Belgian hospitalized COVID-19 patients registered in the Clinical Hospital Surveillance (CHS) and admitted between the 1st of March 2021 and the 28th of March 2022 by the availability of SARS-CoV-2 variant information (variant information confirmed by Whole Genome Sequencing (WGS) available versus no confirmed variant information available) (left y-axis) and the coverage of Belgian hospitalized COVID-19 patients with confirmed variant information, i.e. percentage of patients with available confirmed (by WGS) viral information, among the of Belgian hospitalized COVID-19 patients admitted between the 1st of March 2021 and the 28th of March 2022 (right y-axis). [file 12879_2022_7777_MOESM3_ESM.docx]

#### Additional File 3: Coverage of Belgian hospitalized COVID-19 patients with confirmed SARS-CoV-2 variant information


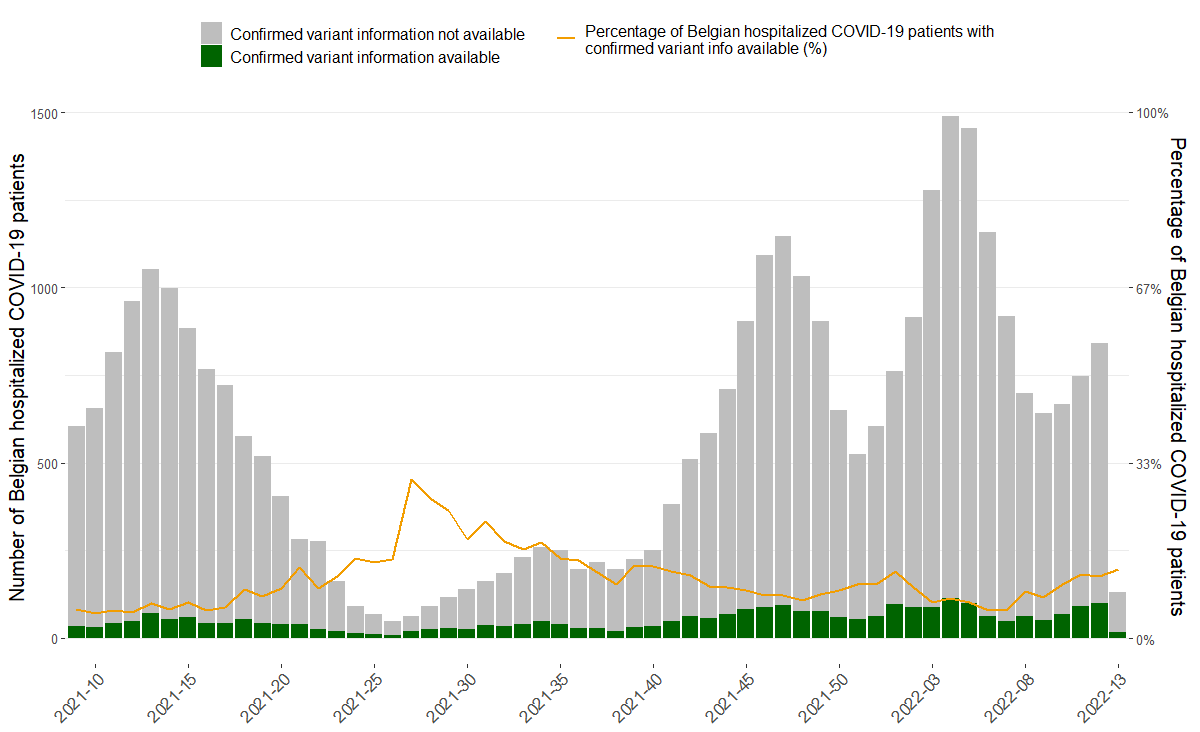


***Figure S2****. The number of Belgian hospitalized COVID-19 patients registered in the Clinical Hospital Surveillance (CHS) and admitted between the 1^st^ of March 2021 and the 28^th^ of March 2022 by the availability of SARS-CoV-2 variant information (variant information confirmed by Whole Genome Sequencing (WGS) available* versus *no confirmed variant information available) (left y-axis) and the coverage of Belgian hospitalized COVID-19 patients with confirmed variant information, i.e. percentage of patients with available confirmed (by WGS) viral information, among the of Belgian hospitalized COVID-19 patients admitted between the 1^st^ of March 2021 and the 28^th^ of March 2022 (right y-axis).*
