# Supplementary material for: Evaluating methodological approaches to assess the severity of infection with SARS-CoV-2 variants: scoping review and applications on Belgian COVID-19 data
Source: BMC Infect Dis. 2022 Nov 11;22:839. doi: 10.1186/s12879-022-07777-6 (PMC9651100; doi:10.1186/s12879-022-07777-6)
Supplement: Supplementary file 4 — Additional file 4: Figure S3. Vaccination status of Belgian hospitalized COVID-19 patients registered in the Clinical Hospital Survey (CHS). (Left) Number of Belgian hospitalized COVID-19 patients with a certain vaccination status over time, 7-day rolling average. (Right) Percentage of Belgian hospitalized COVID-19 patients with a certain vaccination status over time, 7-day rolling average. Periods of dominance of SARS-CoV-2 variants (more than 50% presence in baseline surveillance) are indicated as areas on the plot. Not vaccinated = no dose of a vaccine. Partially vaccinated = one dose of the BNT162b2, mRNA-1273 or NVX-CoV2373 vaccine. Fully vaccinated = one dose of the Ad26.COV2.S vaccine, two doses of the BNT162b2, mRNA-1273 or NVX-CoV2373 vaccine, or a mixture of two doses of the latter three vaccines (= primary vaccination schedule). Fully vaccinated + booster = primary vaccination schedule plus an additional dose of the BNT162b2 or mRNA-1273 vaccine. [file 12879_2022_7777_MOESM4_ESM.docx]

#### Additional File 4: Vaccination status of Belgian hospitalized COVID-19 patients


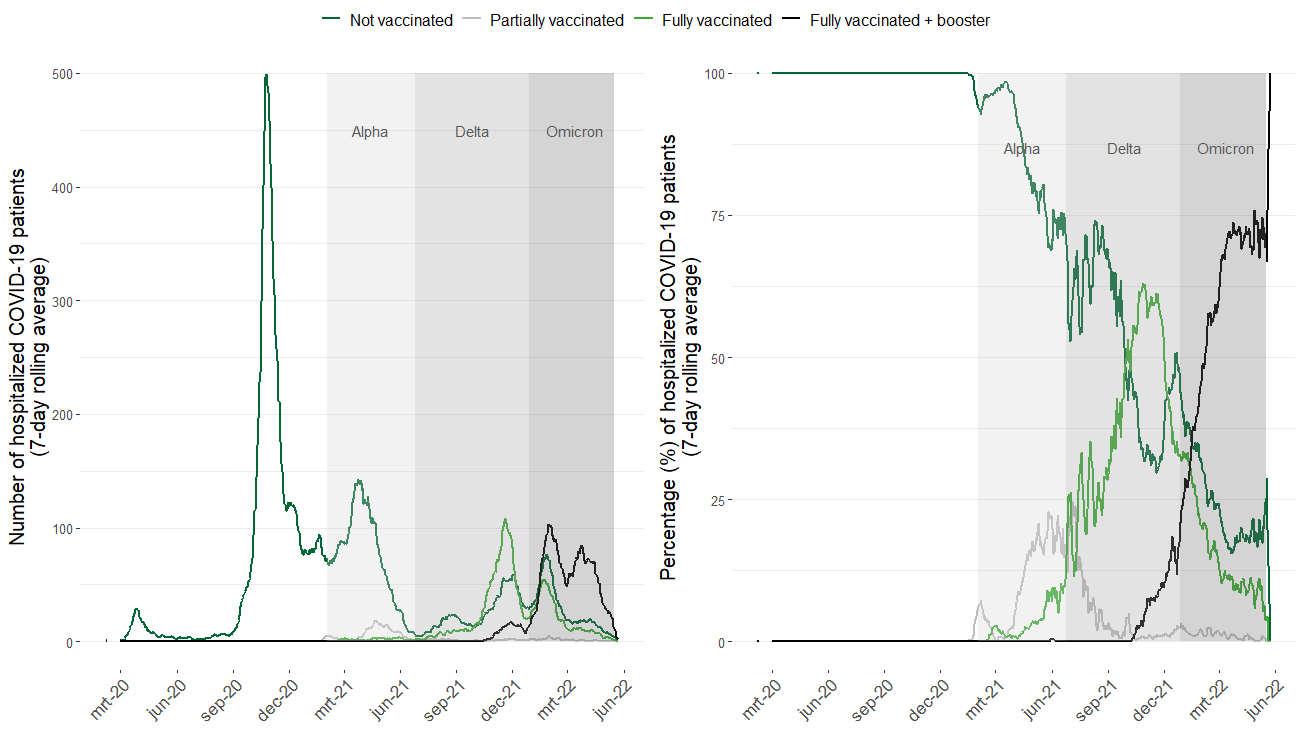


*Figure S3. Vaccination status of Belgian hospitalized COVID-19 patients registered in the Clinical Hospital Survey (CHS). (Left) Number of Belgian hospitalized COVID-19 patients with a certain vaccination status over time, 7-day rolling average. (Right) Percentage of Belgian hospitalized COVID-19 patients with a certain vaccination status over time, 7-day rolling average. Periods of dominance of SARS-CoV-2 variants (more than 50% presence in baseline surveillance) are indicated as areas on the plot. Not vaccinated = no dose of a vaccine. Partially vaccinated = one dose of the BNT162b2, mRNA-1273 or NVX-CoV2373 vaccine. Fully vaccinated = one dose of the Ad26.COV2.S vaccine, two doses of the BNT162b2, mRNA-1273 or NVX-CoV2373 vaccine, or a mixture of two doses of the latter three vaccines (= primary vaccination schedule). Fully vaccinated + booster = primary vaccination schedule plus an additional dose of the BNT162b2 or mRNA-1273 vaccine.*
