# Supplementary material for: Evaluating methodological approaches to assess the severity of infection with SARS-CoV-2 variants: scoping review and applications on Belgian COVID-19 data
Source: BMC Infect Dis. 2022 Nov 11;22:839. doi: 10.1186/s12879-022-07777-6 (PMC9651100; doi:10.1186/s12879-022-07777-6)
Supplement: Supplementary file 6 — Additional file 6: Figure S5. Nursing home residents within the population of Belgian hospitalized COVID-19 patients registered in the Clinical Hospital Survey (CHS). (Left) Number of Belgian hospitalized COVID-19 patients that are nursing home residents, 7-day rolling average. (Right) Percentage of Belgian hospitalized COVID-19 patients that are nursing home residents, 7-day rolling average. Periods of dominance of SARS-CoV-2 variants (more than 50% presence in baseline surveillance) are indicated as areas on the plot. [file 12879_2022_7777_MOESM6_ESM.docx]

#### Additional File 6: Nursing home residents within the population of Belgian hospitalized COVID-19 patients


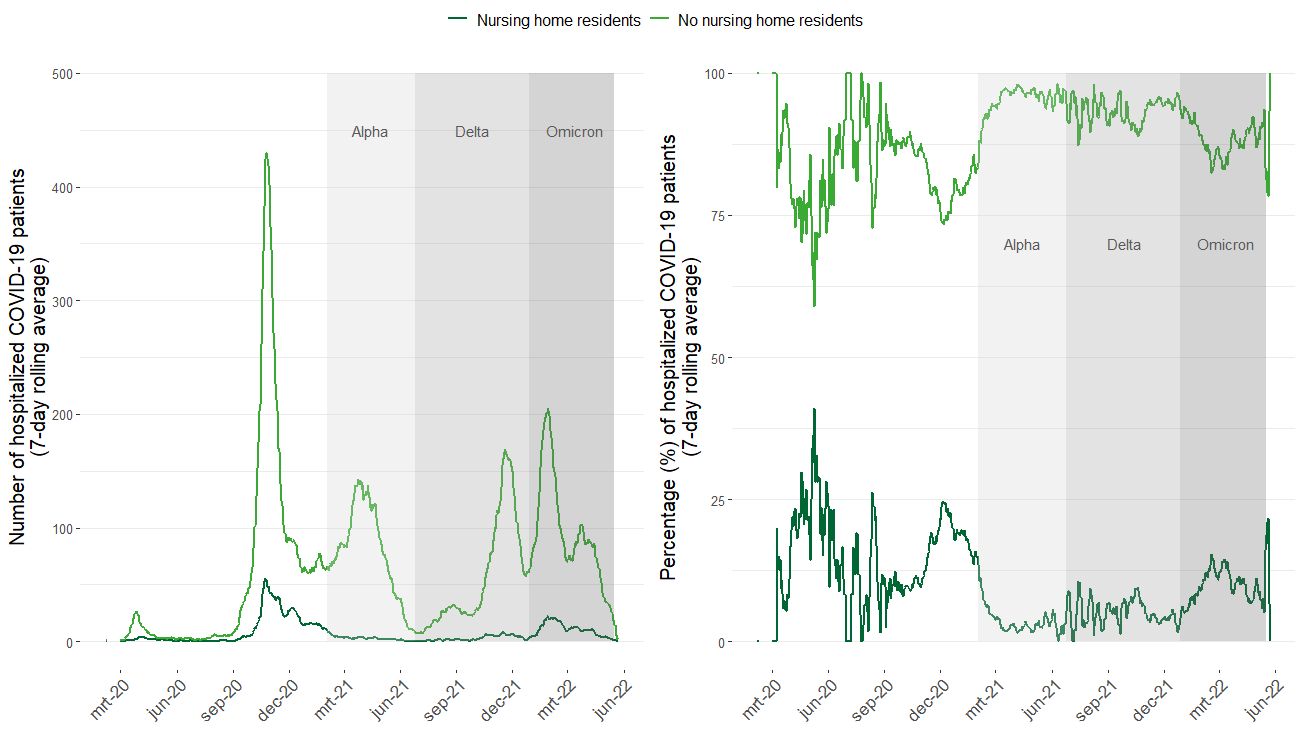


*Figure S5: Nursing home residents within the population of Belgian hospitalized COVID-19 patients registered in the Clinical Hospital Survey (CHS). (Left) Number of Belgian hospitalized COVID-19 patients that are nursing home residents, 7-day rolling average. (Right) Percentage of Belgian hospitalized COVID-19 patients that are nursing home residents, 7-day rolling average. Periods of dominance of SARS-CoV-2 variants (more than 50% presence in baseline surveillance) are indicated as areas on the plot.*
