# Supplementary material for: Evaluating methodological approaches to assess the severity of infection with SARS-CoV-2 variants: scoping review and applications on Belgian COVID-19 data
Source: BMC Infect Dis. 2022 Nov 11;22:839. doi: 10.1186/s12879-022-07777-6 (PMC9651100; doi:10.1186/s12879-022-07777-6)
Supplement: Supplementary file 7 — Additional file 7: Figure S6. (Left) Median age of Belgian hospitalized COVID-19 patients registered in the Clinical Hospital Survey (CHS), 7-day rolling average. (Right) Median of the mean ICU occupancy of Belgian hospitalized COVID-19 patients registered in the CHS, 7-day rolling average. Periods of dominance of SARS-CoV-2 variants (more than 50% presence in baseline surveillance) are indicated as areas on the plot. [file 12879_2022_7777_MOESM7_ESM.docx]

#### Additional File 7: Age and mean ICU occupancy of Belgian hospitalized COVID-19 patients

####
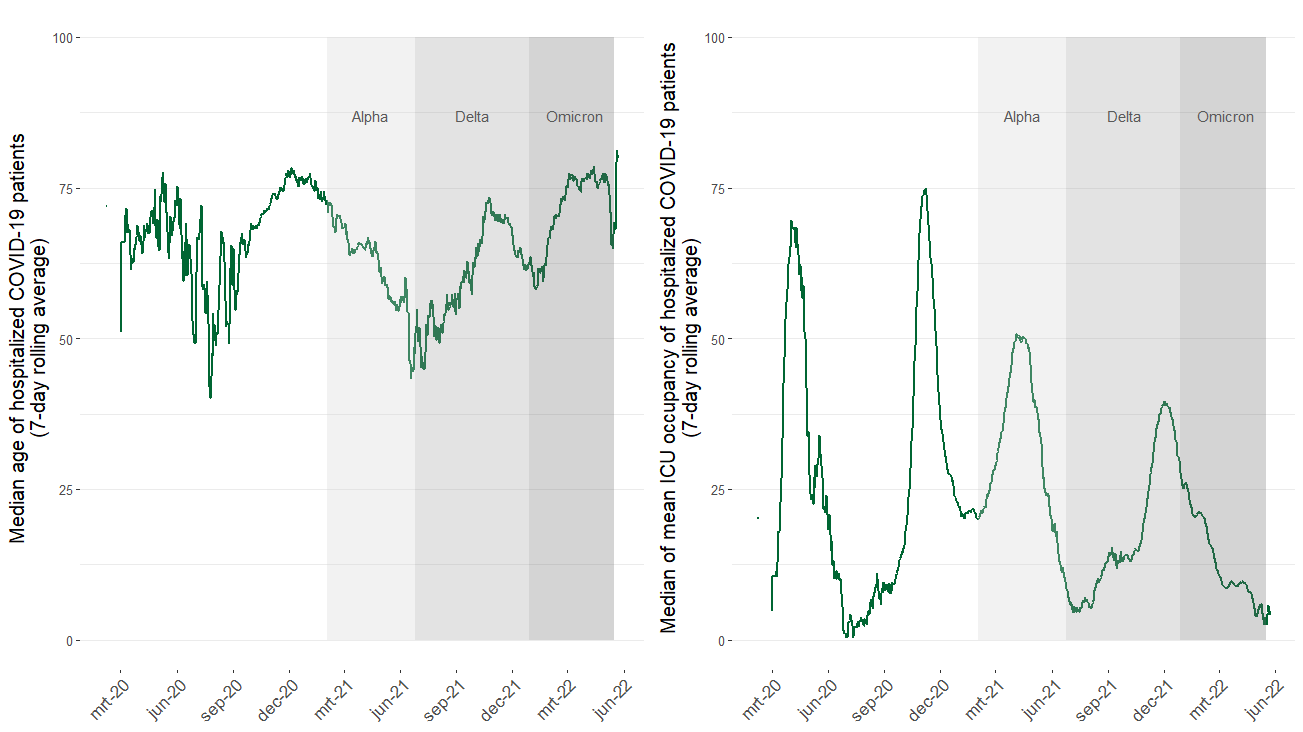


*Figure S6. (Left) Median age of Belgian hospitalized COVID-19 patients registered in the Clinical Hospital Survey (CHS), 7-day rolling average. (Right) Median of the mean ICU occupancy of Belgian hospitalized COVID-19 patients registered in the CHS, 7-day rolling average. Periods of dominance of SARS-CoV-2 variants (more than 50% presence in baseline surveillance) are indicated as areas on the plot.*
